# Mapping Important Shark and Ray Areas (ISRAs) in the Central and South American Pacific: Existing knowledge and data needs

**DOI:** 10.1371/journal.pone.0322445

**Published:** 2025-05-07

**Authors:** Emiliano García-Rodríguez, Adriana Gonzalez-Pestana, Ryan Charles, Marta D. Palacios, Giuseppe Notarbartolo di Sciara, Joanna Alfaro-Shigueto, Cristopher G. Avalos-Castillo, Elpis J. Chávez, Mario Espinoza, Ana Hacohen-Domené, Alex R. Hearn, Felipe Galván-Magaña, James T. Ketchum, Frida Lara-Lizardi, Jorge Manuel Morales-Saldaña, Naití Morales Serrano, Paola A. Mejía-Falla, Andrés F. Navia, César R. Peñaherrera-Palma, Francisco Polanco-Vásquez, Yehudi Rodríguez-Arriatti, Luz E. Saldaña-Ruiz, Oscar Sosa-Nishizaki, Ximena Velez-Zuazo, Rima W. Jabado

**Affiliations:** 1 Department of Biological Oceanography, Centro de Investigación Científica y de Educación Superior de Ensenada, Ensenada, Baja California, Mexico; 2 International Union for Conservation of Nature Species (IUCN), Species Survival Commission (SSC) Shark Specialist Group, Dubai, United Arab Emirates; 3 Carrera de Biología Marina, Universidad Científica del Sur, Lima, Peru; 4 Mobula Conservation, La Paz, Mexico; 5 Tethys Research Institute, Milano, Italy; 6 IUCN Joint Species Survival Commission (SSC)/World Commission on Protected Areas (WCPA) Marine Mammal Protected Areas Task Force, Gland, Switzerland; 7 ProDelphinus, Lima, Peru; 8 Centro de Estudios del Mar y Acuicultura, Universidad de San Carlos de Guatemala, Guatemala, Guatemala; 9 Centro Rescate de Especies Marinas Amenazadas, San José, Costa Rica; 10 MigraMar, Bodega Bay, California, United States of America; 11 Centro de Investigación en Ciencias del Mar y Limnología, Universidad de Costa Rica, San Pedro, San José, Costa Rica; 12 Escuela de Biología, Universidad de Costa Rica, San Pedro, 11501 San José, Costa Rica; 13 Biology Department, Universidad del Valle de Guatemala, GuatemalaGuatemala; 14 School of Biological and Environmental Sciences, COCIBA, Universidad San Francisco de Quito, Quito, Ecuador; 15 Instituto Politécnico Nacional, Centro Interdisciplinario de Ciencias Marinas, La Paz, Baja California Sur, Mexico; 16 Pelagios Kakunjá, La Paz, Baja California Sur, México; 17 Centro de Investigaciones Biológicas del Noroeste, La Paz, Baja California Sur, México; 18 ORGCAS, La Paz, Baja California Sur, Mexico; 19 Smithsonian Tropical Research Institute, Balboa, Republic of Panama,; 20 Department of Biology, McGill University, Montreal, Quebec, Canada; 21 Center for Ecology and Sustainable Management of Oceanic Islands (ESMOI), Universidad Católica del Norte, Coquimbo, Chile; 22 Instituto de Fomento Pesquero, Valparaíso, Chile; 23 Wildlife Conservation Society, Colombia, Cali, Colombia; 24 Fundación colombiana para la investigación y conservación de tiburones y rayas, Cali, Colombia; 25 Wildlife Conservation Society, Guatemala Program, Flores, Petén, Guatemala; 26 Centro de Estudios del Mar y Acuicultura, Universidad de San Carlos de Guatemala, Ciudad Universitaria, Zona 12, Guatemala; 27 Shark Defenders, Panama City, Panama; 28 Secretaría de Ciencia, Humanidades, Tecnología e Innovación (SECIHTI), Ciudad de Mexico, Mexico; 29 Smithsonian National Zoological Park and Conservation Biology Institute, Washington, District of Columbia, United States of America; 30 College of Science and Engineering, James Cook University, Townsville, Qld, Australia; 31 Elasmo Project, Dubai, United Arab Emirates.; University of Messina, ITALY

## Abstract

Identifying critical habitats is key to the conservation and recovery of threatened species. A third of chondrichthyans (sharks, rays, and chimaeras) are threatened with extinction but robust biological and ecological information to delineate critical habitats for many species remains limited. Here, we investigated (1) research outputs and trends across the Central and South American Pacific region to determine whether sufficient information was available to identify critical habitats; (2) whether regional Important Shark and Ray Areas (ISRAs) were spatially representative; (3) what species and which ecological traits were most commonly used in the delineation of critical habitats; and (4) discuss how ISRAs can inform research priorities and area-based management in support of chondrichthyan conservation. Sixty-five ISRAs were identified for 97 of 190 chondrichthyan species occurring in the region (51%). Across key life-history processes, reproductive areas were most identified (n = 50). Of 821 published studies (2,160 entries), 31.48% (28% entries) primarily focused on fisheries and 48.51% included enough information to inform the ISRA process. Most (58.98%) of these studies originated from Mexico (n = 342, 744 entries) and Ecuador (n = 147, 276 entries). France and Honduras had the least regional research outputs relevant to apply the ISRA Criteria. Significant ecological data gaps were identified in oceanic (including areas beyond national jurisdiction), deepwater (>200 m), and along the southern part of the region (i.e., southern Chile). Deepwater species, chimaeras, and 21% of threatened species had knowledge gaps that did not allow the identification of ISRAs. If area-based management decisions in this region were based on ISRAs, and effectively implemented and enforced, diversity hotspots and at least 97 species could receive protection, including 79% of threatened species and 54% of those considered range-restricted. Increased monitoring and research efforts, with a corresponding increase in funding to fill existing gaps is key to support the identification of important habitats across this region.

## Introduction

Critical habitats are areas that support life-history characteristics and vital functions of species, such as reproduction, feeding, and movement [1,2]. These areas are often considered in national legislative frameworks as key elements to aid in the conservation and recovery of imperiled species [3,4]. Their identification therefore needs to be based on robust biological and ecological data [5]. However, the information needed to accurately identify these habitats is not always readily available, hampering the effectiveness of conservation measures and efforts (e.g., action plans, management strategies, and protected area delineation).

Chondrichthyans (sharks, rays, and chimaeras) are facing a global extinction crisis. Of the ~ 1,250 species described, over one-third (37%) are considered threatened with extinction according to the IUCN Red List of Threatened Species, making them the second most threatened group of vertebrates after amphibians [6]. Overfishing is the main threat, followed by habitat degradation and to a lesser extent climate change and pollution [6,7]. Due to their elevated extinction risk, protecting their critical habitat from these threats is urgently needed to reduce mortality and ensure the recovery of this ecologically important and diverse group of fishes [2].

Among aquatic taxa, chondrichthyans have been relatively poorly studied compared to other vertebrates [6,8–10]. In certain cases, this has limited the evaluation of population trends along with the identification of critical habitats [11,12]. Indeed, research efforts focused on these species have often been unevenly distributed across time, species, regions, and topics [13,14]. This has led to knowledge gaps and biased research which makes it difficult to interpret studies and draw conclusions to inform policy and conservation actions [13,15]. Many species remain understudied, and knowledge of basic biology and ecology is lacking even for species occurring in habitats with a relatively high research effort [15,16]. Assessing and recognizing these differences in scientific focus and outputs across species and locations is key to improving our knowledge, understanding research needs, and prioritizing management actions [13,17].

In response to the rapid and increasing loss of biodiversity, the Kunming-Montreal Global Biodiversity Framework (GBF) was adopted by many governments worldwide to ensure the conservation and sustainable use of biodiversity to benefit the planet and those who inhabit it [18]. Specifically, Target 3 commits countries to protecting at least 30% of their national terrestrial, inland waters, and marine areas by 2030. A key aspect to guarantee the success of this framework is to focus on the protection of areas of particular importance to decrease the loss of biodiversity and maintain ecosystem function [19]. The Important Shark and Ray Areas (ISRA) approach was developed to map important habitats for key life history processes of chondrichthyans across all global waters [2]. The ISRA framework identifies ‘discrete, three-dimensional portions of habitat important for one or more shark, ray, or chimaera species, that are delineated and have the potential to be managed for conservation’ [2]. The ISRA Criteria consider the vulnerability, range restriction, diverse life-histories, and special attributes of chondrichthyans and can be applied to all environments where they occur. Information derived from this process provides governments and policymakers access to scientifically defined areas that can help them advance actions to conserve these species [20]. The ISRA identification process can also help identify regional knowledge gaps and priority research areas needed to support the delineation of critical habitats.

In 2022, this process was applied to the Central and South American Pacific region and 65 ISRAs were identified [21]. This region harbors 96 shark, 83 ray, and 11 chimaera species representing ~15% of the global reported diversity. In addition, it is a hotspot for endemic species, data-deficient species, evolutionary distinctiveness, and is characterized by high functional richness [6,22,23]. Overall, 37% of the species (n = 72) reported from this region are considered range-restricted according to the ISRA Criteria (i.e., species whose distribution is limited to one or two adjacent Large Marine Ecosystems) and 39% (n = 75) are threatened with extinction according to the IUCN Red List [24].

Here, we investigated (1) research outputs and trends across in the Central and South American Pacific region to determine whether sufficient information was available to identify important habitats for chondrichthyans; (2) whether ISRAs in this region were spatially representative; (3) what species and which ecological traits were most commonly used in the delineation of critical habitats; and (4) discuss how the identification of ISRAs can inform research priorities and area-based management to support the conservation of this taxon group.

## Methods

### Study area

**Fig 1:**
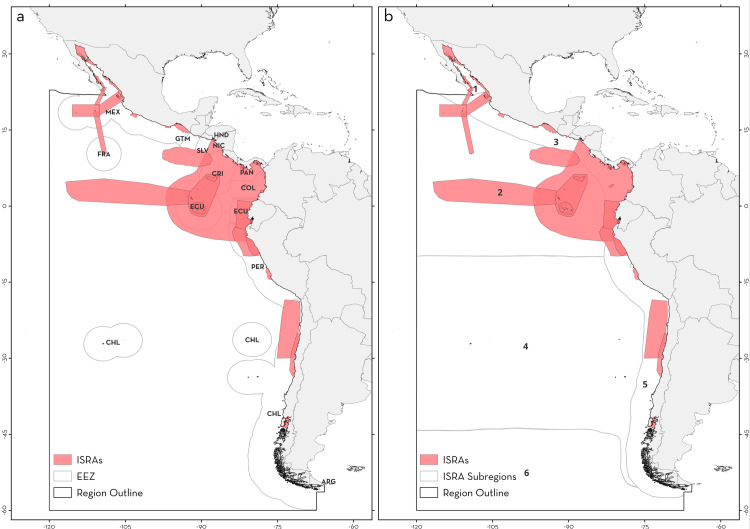
Important Shark and Ray Areas (ISRA) identified in the Central and South American Pacific region. (a) Three-letter codes inside the Exclusive Economic Zones (EEZ) represent the corresponding jurisdictions. CHL: Chile, COL: Colombia, CRI: Costa Rica, ECU: Ecuador, FRA: France, GTM: Guatemala, HND: Honduras, MEX: Mexico, NIC: Nicaragua, PAN: Panama, PER: Peru, SLV: El Salvador. (b) Numbers represent each subregion within the Central and South American Pacific region. 1: Gulf of California, 2: Offshore Eastern Pacific, 3: Pacific Central American Coastal, 4: Southeast Pacific, 5: Humboldt Current, 6: South American Southern Ocean. EEZ boundaries were reprinted from Flanders Marine Institute (2023) Maritime Boundaries Geodatabase: Maritime Boundaries and Exclusive Economic Zones (200NM), version 12. Available online at https://www.marineregions.org/. https://doi.org/10.14284/632 under a CC BY 4.0 international license.

The Central and South American Pacific region as considered in this study extends from the Gulf of California, Mexico to the tip of southern Chile, covering the Exclusive Economic Zones (EEZ) of 12 countries and Areas Beyond National Jurisdiction (ABNJ; Fig. 1). This includes oceanic islands (e.g., Revillagigedo Archipelago, Clipperton Atoll, Cocos Island, Malpelo Island, Galápagos Archipelago, and Rapa Nui) and the central and southern Eastern Pacific oceanic area [21]. Three Large Marine Ecosystems (LMEs; Gulf of California, Pacific Central-American Coastal, and Humboldt Current [25]), and five Biogeographic Marine Realms (Tropical Eastern Pacific, Southeast Pacific, Offshore Mid Eastern Pacific, Chile, and Southern Ocean [26]) are included within this region (Fig. 1). To examine critical habitats for chondrichthyans, the Central and South American Pacific region was divided into six subregions: Gulf of California, Offshore Eastern Pacific, Pacific Central American Coastal, Southeast Pacific, Humboldt Current, and South American Southern Ocean. These subregions were delineated based on the boundaries of Large Marine Ecosystems (within coastal areas) and biogeographic marine realms (in oceanic areas; [26–28]).

### Delineation of important Shark and Ray areas

An expert workshop with representatives from jurisdictions in the region was held in Bogotá, Colombia in October 2022 [21]. Participants proposed preliminary Areas of Interest considering various life-history processes for chondrichthyans based on contemporary (less than 20 years old; 2002–2022) data (e.g., from research surveys, local ecological knowledge; Table 1). The four ISRA Criteria (divided into seven sub-criteria) were considered: A - Vulnerability (i.e., species assessed as Critically Endangered, Endangered, or Vulnerable according to the IUCN Red List), B - Range Restricted (i.e., species whose distribution is limited to one or two adjacent Large Marine Ecosystems), C - Life-history (i.e., reproduction, feeding, resting, movement, or undefined aggregations), and D - Special Attributes (i.e., distinctiveness which is related to distinct biological, behavioral, or ecological characteristics; and diversity which is related to species richness) ([2] Table 1). Species confirmed to occur regularly and/or predictably in an area and satisfying one or more ISRA Criteria are defined as ‘Qualifying Species’. Species whose presence is regular or predictable but do not satisfy any of the ISRA Criteria are defined as ‘Supporting Species’ [24]. ISRAs were delineated when sufficient information was available to meet one or more ISRA Criteria and after undergoing an external review process. Details of the ISRA process can be found in [2] and [24].

**Table 1:** Types of biological and ecological data sources and research methods used to support the application of the ISRA Criteria. YOY (young-of-the-year), LEK (local ecological knowledge), eDNA (environmental DNA), BRUVS (baited remote underwater video systems), ROV (remotely operated underwater vehicle), DOV (diver operated video), and UVC (underwater visual census).

ISRA Criteria	Type of biological/ecological data needed	Research methods and technologies
**A - Vulnerability** *Criterion A can only be applied with an additional criterion.*	**Vulnerability:** extinction risk status of the species	• IUCN Red List of Threatened Species (Critically Endangered, Endangered, or Vulnerable) • Regional or national regulatory and legal frameworks for assessing species extinction risk (if a higher risk status is assigned than the IUCN Red List)
**B - Range Restricted**	**Demographic:** number of individuals, sightings or records	• Direct observations (LEK, citizen science, or researchers) • Catch-per-unit-effort and fisheries landings • Count surveys (sightings-per-unit-effort) • eDNA • BRUVS • ROV, DOV, UVC • Mark-recapture surveys
**C1 - Reproductive Areas**	**Demographic:** number of individuals by size (total length or disc width), presence of neonates/young-of-the-year (YOY), and/or by presence of umbilical or mating scars and distended abdomen, aborted pups or egg cases **Behavioral:** number of observations of mating or courtship behavior **Habitat use:** residency in the area by neonates and/or YOY	• Direct observations (LEK, citizen science, or researchers) • Catch-per-unit-effort and fisheries landings (pregnant, neonates, YOY, or egg cases) • Count surveys (sightings-per-unit-effort) • Animal mounted cameras • Aerial surveys • Autonomous underwater stationary cameras • Molecular genetics for natal philopatry • Underwater portable ultrasonography • ROV, DOV, UVC
**C2 - Feeding Areas**	**Behavioral:** number of observations of feeding behavior **Trophic:** number of individuals with stomach contents or isotopic signatures of local prey **Environmental:** high productivity (e.g., upwelling) or high abundance of prey	• Direct observations (LEK, citizen science, or researchers) • Stomach content analysis • Isotope/fatty acid analysis • Aerial surveys • Animal mounted cameras • Autonomous underwater stationary cameras • Nourishment condition indicators • Accelerometers • Prey surveys • Oceanographic conditions • ROV, DOV, UVC
**C3 - Resting Areas**	**Behavioral:** number of observations of resting behavior **Environmental:** oceanographic and bathymetric features	• Direct observations (LEK, citizen science or researchers) • Autonomous underwater stationary cameras • Accelerometers • Animal mounted cameras • Aerial surveys • BRUVS, ROV, and DOV
**C4 - Movement Areas**	**Spatial:** number of individuals following similar spatial movement patterns	• Satellite tracking • Acoustic tracking • Photo-identification
**C5 - Undefined Aggregations**	**Demographic:** number of individuals interacting as a group	• Direct observations (LEK, citizen science, or researchers) • Catch-per-unit-effort • Count surveys (sightings-per-unit-effort) • Acoustic telemetry • Aerial surveys • Animal mounted cameras • Autonomous underwater stationary cameras • BRUVS, ROV, and DOV • Animal-borne acoustic proximity receivers • Sound recording (rays) • High-resolution acoustic cameras
**D1 - Distinctiveness**	**Behavioral:** number of observations of behavior	• Direct observations (LEK, citizen science, or researchers) • Count surveys (sightings-per-unit-effort) • Animal mounted cameras • Aerial surveys • Autonomous underwater stationary cameras - • Stable Isotopes/Fatty acid analysis • Stomach content analysis
**D2 - Diversity**	**Diversity**: number of species regularly and/or predictably occurring in the area	• The ISRA regional threshold of number of species is met/exceeded

### Spatial distribution of ISRAs and ISRA criteria met

A descriptive analysis of the 65 ISRAs delineated in this region was undertaken. This allowed to (1) identify the most common type of biological or ecological information used to support the identification of an ISRA, and (2) examine which criteria or sub-criteria were most frequently applied to support the identification of ISRA in each of the six subregions.

### Qualifying Species description

The number of Qualifying Species, their taxonomic order, and extinction risk status according to the IUCN Red List (data extracted in August 2023; www.iucnredlist.org)[29,30] were analyzed to determine which species had enough information to apply the ISRA Criteria and to explore regional knowledge gaps at the family level.

### Overlap of ISRAs with the distribution of range-restricted species and diversity hotspots

Maps representing species richness for (1) range-restricted species and (2) diversity hotspots in the Central and South American Pacific region were created based on published IUCN Red List range maps [29,30]. Species distribution maps were rasterized and merged with a 0.1 km resolution using ArcGIS Pro 3.3 [31]. Only the extant distribution of species was considered.

ISRAs identified by applying Sub-criterion D2 - Diversity are those meeting a regional threshold of species richness. The regional threshold is based on a relative numerical assessment, depending on the total chondrichthyan diversity within a region. To define this threshold, species richness maps (based on the IUCN Red List distribution maps) were created and consulted to estimate the maximum diversity in the region within a single cell (0.1 km^2^). The final regional threshold was set based on a value of 30% of species contained within the cell with the highest species richness, which was 57 species for this region [24]. The diversity threshold for the Central and South American Pacific region was set at 17 species (30% of the 57 species), representing 9% of total regional species richness for the region [30].

We estimated the spatial overlap between the distribution of at least one range-restricted species and all ISRAs in the region. We also examined the spatial overlap of the distribution of at least one range-restricted species with ISRAs identified using Criterion B - Range Restricted. Finally, we investigated the overlap between diversity hotspots (areas with a diversity of regularly occurring species equal to or larger than the threshold for this region), all ISRAs, and ISRAs delineated using Sub-criterion D2 – Diversity. All analyses were done using the *Overlay* tool in ArcGIS Pro 3.3 (ESRI Inc 2024]. Two ISRAs (Eastern Tropical Pacific Marine Corridor ISRA and Gulf of California-Revillagigedo-Clipperton Migration Corridor ISRA), delineated based on the large-scale movements of highly migratory sharks (e.g., Whale Shark *Rhincodon typus*), were not included in the analysis because their boundaries overlapped with other smaller ISRAs, and they did not meet the Range Restricted or the Diversity criteria.

### Research trends and gaps analysis

To explore research outputs related to chondrichthyans in the region, a review of available regional references was undertaken. This was performed by searching for keywords in English and Spanish in web engines (i.e., Google Scholar, Web of Science) and databases from academic institutions and environmental and fishing authorities (e.g., thesis from the Universidad San Francisco de Quito in Ecuador or reports from the Mexican Institute for Research in Sustainable Fisheries and Aquaculture). The keywords used were ‘shark/tiburon’, ‘batoid/batoideo’, ‘ray/raya/mantarraya’, ‘chimaera/quimera’, ‘elasmobranch/elasmobranquio’, and ‘chondrichthyan/condrictio’ in addition to terms related to the ISRA Criteria: ‘habitat/hábitat’, ‘reproduction/reproducción’, ‘feeding/alimentación’, ‘resting/descanso’, ‘movement/movimiento’, and ‘aggregation/agregación’. Furthermore, references were directly gathered from regional workshop participants and contributors to the ISRA process. References collated included scientific papers, books (including book chapters), and grey literature (e.g., technical reports and unpublished thesis). To avoid including data from habitats that were important in the past but currently are not (e.g., due to population reductions or habitat loss), only contemporary information (<20 years old) was considered. Nevertheless, we also included selected references older than 20 years when they contained detailed information used to satisfy the ISRA Criteria and could supplement contemporary information.

We explored the historical trends of publications at six different levels and Sankey plots were created to visually illustrate them using the *ggsankey* package in the R statistical software (v 4.2.3; [32]). The levels considered for all collected references were:

- **Year of publication:** references were categorized in five-year intervals (prior to 2002 and 2002–2007: 2007–2012; 2013–2017; and 2018–2022).
- **Type of document:** peer-reviewed articles (including pre-prints), theses across all academic levels, books (including book chapters), and technical reports;
- **Research topic:** nine topics were defined to encompass the whole range of research on chondrichthyan species within the region and to match the ISRA Criteria as closely as possible (Table 2).
- **Subregion**: based on the six subregions defined for this region;
- **Jurisdiction;**
- **ISRA application:** if the reference was used during the regional ISRA delineation process or not;
- **Taxa:** sharks, rays, or chimaeras; and
- **Type of species according to the ISRA process:** Qualifying Species or Supporting Species.

**Table 2:** Type of studies included in research topics focused on chondrichthyans in the Central and South American Pacific region.

Research topic	Type of studies included	Number of entries
**Age and growth**	- Age estimations - Length and weight estimations	96
**Diversity**	- Species checklists - Species composition and abundance estimates (i.e., fisheries independent data)	357
**Fisheries and population assessment**	- Catch and landings descriptions (i.e., fisheries dependent data) - Demographic studies - Vulnerability analyses - Stock assessments	610
**Human dimensions**	- Economic value of chondrichthyans by extractive and non-extractive activities - Human values and attitudes toward chondrichthyans	53
**Reproductive biology**	- Description of reproductive processes - Nursery area identification	204
**Spatial ecology**	- Habitat use and preferences - Movement and migration (based on tagged individuals) - Range extensions - Relationship of distribution with environmental factors	376
**Taxonomy**	- New species descriptions - Stock structure based on genetic analysis	113
**Trophic ecology**	- Direct observations of feeding events - Fatty acids - Stable isotope analysis - Stomach content analysis	204
**Other**	- Morphological analysis - Parasite studies - Pollutant analyses - Physiology studies	133

Some references spanned different jurisdictions, topics, and multiple species. To use in the analysis, multiple entries were created for a single reference (e.g., one study undertaken in both Costa Rica and Ecuador focused on the general biology of sharks and covered the topics of trophic ecology, age and growth, and reproductive biology).

## Results

### ISRA Criteria met

Of the 65 ISRAs identified in the Central and South American Pacific region, the most common criteria used for delineation were A - Vulnerability (n = 61, 93.84%) and C1 - Reproductive Areas (n = 50, 76.92%), followed by C2 - Feeding Areas (n = 28, 43.07%), B - Range Restricted (n = 27, 41.53%), C4 - Movement Areas (n = 22, 33.84%), C5 - Undefined Aggregations (n = 18, 27.69%), and D2 - Diversity (n = 13, 20%). The least applied ISRA Criteria were C3 - Resting Areas (n = 8, 12%) and D1 - Distinctiveness (n = 6, 9.23%).

Ninety-seven chondrichthyan species were included as Qualifying Species. As expected, based on the regional diversity threshold, the criterion with the highest number of Qualifying Species was D2 - Diversity (n = 79, 81.44%). It was followed by C1 - Reproductive Areas (n = 56, 57.73%; Table 3). All ISRA Criteria were applied to all sharks and rays, except C3 - Resting for rays. Chimaeras were only included in the B - Range Restricted and D2 - Diversity criteria (Table 3).

**Table 3:** Number of shark, ray, and chimaera species considered as Qualifying Species based on each Important Shark and Ray Areas (ISRA) Criteria in the Central and South American Pacific region. Numbers in parentheses represent the percentage of species meeting the ISRA Criteria relative to the 190 species reported for this region (except for Criterion A – Vulnerability).

ISRA Criteria	Sharks	Rays	Chimaeras	All
A - Vulnerability	33 (78.57)	26 (81.25)	0 (0)	59 (78.67)
B - Range Restricted	14 (63.64)	23 (50.00)	2 (50.00)	39 (54.17)
C1 - Reproductive Areas	29 (29.79)	27 (30.21)	0 (0)	56 (29.79)
C2 - Feeding Areas	20 (20.83)	22 (26.51)	0 (0)	42 (22.34)
C3 - Resting Areas	2 (2.08)	0 (0)	0 (0)	2 (1.06)
C4 - Movement Areas	13 (13.54)	4 (4.82)	0 (0)	17 (9.04)
C5 - Undefined Aggregations	7 (7.29)	3 (3.61)	0 (0)	10 (5.32)
D1 - Distinctiveness	7 (7.29)	1 (1.20)	0 (0)	8 (4.26)
D2 - Diversity	38 (39.58)	39 (46.99)	2 (18.18)	79 (42.02)

### Spatial distribution of ISRAs

**Fig 2:**
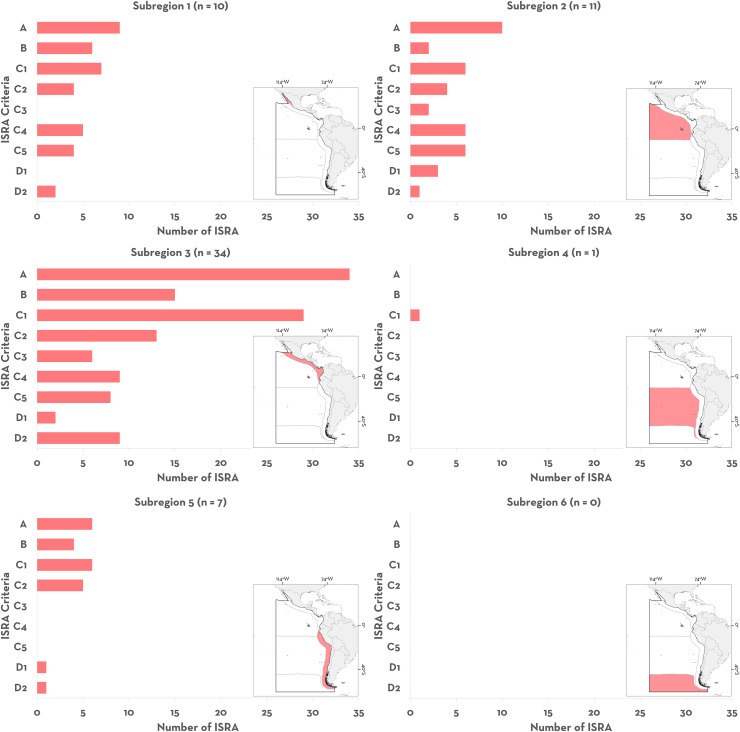
The six subregions of the Central and South American Pacific with information on the number of ISRA (in parenthesis) delineated and the ISRA Criteria applied in each. Two movement areas spanned multiple subregions and were not included in this figure. ISRA Criteria are: A: Vulnerability; B: Range Restricted; C1: Reproductive Areas; C2: Feeding Areas; C3: Resting Areas; C4: Movement Areas; C5: Undefined Aggregations; D1: Distinctiveness; D2: Diversity.

From a subregional perspective, the Pacific Central American Coastal had the largest number of ISRAs identified (n = 34, 52.30%), followed by the Offshore Mid Eastern Pacific (n = 11, 16.92%), the Gulf of California (n = 10, 15.38%), and the Humboldt Current (n = 7, 10.761%; Fig. 2). Only one ISRA was identified in the Southeast Pacific subregion, and none in the South American Southern Ocean. Two areas spanned multiple subregions, one including the Gulf of California, the Offshore Eastern Pacific, and Pacific Central American Coastal, and the other the Offshore Eastern Pacific and the Pacific Central American Coastal (Fig. 1). The percentage of surface area covered by ISRAs (range of 0.38–3,034,220.26 km^2^ including C4 - Movement Areas; 0.38–148,382 km^2^ excluding C4 - Movement Areas) was higher in the Gulf of California (97,535.5 km^2^; 44.1%), followed by the South American Southern Ocean (683,246.4 km^2^; 26.8%), Offshore Eastern Pacific (2,225,198.3 km^2^; 20.1%), Pacific Central American Coastal (380,353 km^2^; 19.1%), and Southeast Pacific (0.4 km^2^; < 0.001%). In accordance with the general trend, C1 - Reproductive Areas was the most frequently applied criterion for identifying ISRAs across subregions (Fig 2). In addition, ISRAs delineated by applying criterion B - Range Restricted were more frequent in the Gulf of California (n = 6, 60%) and the Pacific Central American Coastal (n = 15, 44.11%) subregions, while C2 - Feeding Areas was also frequent in the latter (n = 13, 38.23%). In the Offshore Eastern Pacific, ISRAs identified as C4 - Movement and C5 - Undefined Aggregations (n = 6, 17.64%) were as common as areas identified for C1 - Reproductive Areas (Fig. 2, S1 Fig).

### Qualifying species and IUCN red list of threatened species status

ISRAs were delineated for 97 (51%) of the 190 chondrichthyans regularly and/or predictably occurring in the Central and South American Pacific region. These 97 Qualifying Species comprised 48 shark, 47 ray, and two chimaera species. Twenty-six (42%) Qualifying Species were included in only one ISRA, 63 (65%) were included in less than six ISRAs, 23 species (24%) were included in 6–10 ISRAs, and only 11 species (11%) were included in more than ten ISRAs.

Of the 10 Qualifying Species most frequently included in ISRAs, seven were sharks and three were rays (S1 Table). Four species were included in more than 20 ISRAs: Scalloped Hammerhead *Sphyrna lewini* (n = 39, 60%), Silky Shark *Carcharhinus falciformis* (n = 24, 36.92%), Blacktip Shark *Carcharhinus limbatus* (n = 22, 33.85%), and Whale Shark (n = 21, 32.31%). The remaining six most frequent Qualifying Species were included in less than 20 ISRAs: Oceanic Manta Ray *Mobula birostris* (n = 17, 26.15%), Longtail Stingray *Hypanus longus* (n = 13, 20.00%), Bull Shark *Carcharhinus leucas* (n = 13, 20.00%), Pelagic Thresher *Alopias pelagicus* (n = 12, 18.46%), Pacific Sharpnose Shark *Rhizoprionodon longurio* (n = 12, 18.46%), and Pacific Eagle Ray *Aetobatus laticeps* (n = 12, 18.46%).

**Fig 3:**
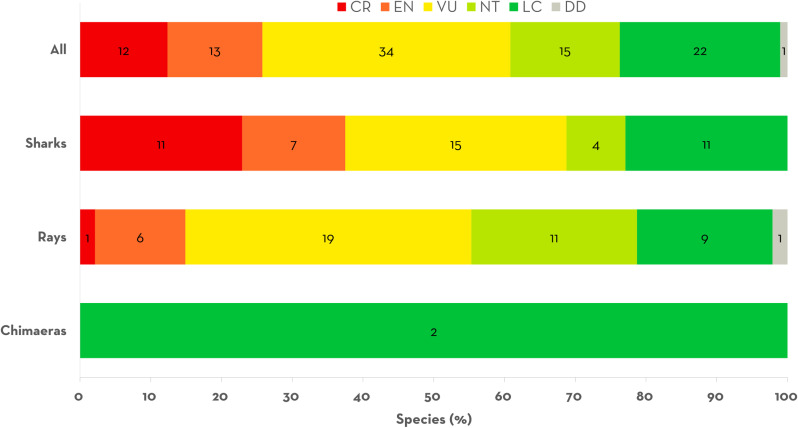
IUCN Red List of Threatened Species extinction risk status for the 75 Qualifying Species in Important Shark and Ray Areas in the Central and South American Pacific. CR: Critically Endangered; EN: Endangered; VU: Vulnerable, NT: Near Threatened; LC: Least Concern; DD: Data Deficient.

Of the 190 species reported for this region, 75 (39.47%) are considered threatened with extinction (42 shark, 32 ray, and 1 chimaera species) and the majority of them (n = 59; 78.66%) were included as Qualifying Species. Among these threatened species, 33 sharks (78.57%) and 26 rays (81.25%) were Qualifying Species. Two (18.18%) of the 11 chimaera species occurring in the region were also Qualifying Species (Fig. 3). All Critically Endangered sharks in this region (n = 11) were Qualifying Species (Fig. 3). Most species assessed as Endangered (n = 13, 72.22%) or Vulnerable (n = 34, 75.55%) were also Qualifying Species. Of the 101 species assessed as Near Threatened (n = 24) or Least Concern (n = 77), the majority could not be considered as Qualifying Species (63.36%). Only one (7.69%) of the 13 Data Deficient species, the Banded Guitarfish *Zapteryx exasperata*, was included as a Qualifying Species.

**Fig 4:**
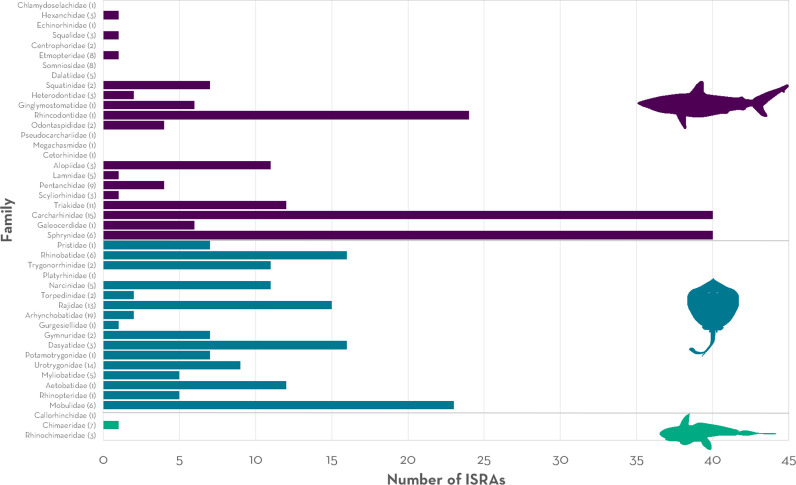
Shark, ray, and chimaera families included as Qualifying Species within an Important Shark and Ray Area (ISRAs) in the Central and South American Pacific region. Numbers in parentheses indicate the number of species for each family occurring in the Central and South American Pacific.

Except for bramble sharks (Echinorhiniformes), all chondrichthyan orders found globally were included in ISRAs as Qualifying Species. Of the 40 families occurring in the region, species from 30 families (82%) were included as Qualifying Species (Fig. 4). All but one ray family (Platyrhinidae), and one of three families of chimaeras (Chimaeridae) were represented in ISRAs. For sharks, 66% of families (n = 16) were represented as Qualifying Species. Shark families included in many ISRAs (>15) were more likely to be coastal (e.g., Carcharhinidae, Sphyrnidae) and pelagic (e.g., Rhincodontidae, Alopiidae), while demersal and deepwater families were poorly represented (e.g., Dalatiidae) (Fig. 4). For rays, pelagic families (e.g., Mobulidae) were included in a larger number of ISRAs while most of the other families were equally represented (Fig. 4).

**Fig 5:**
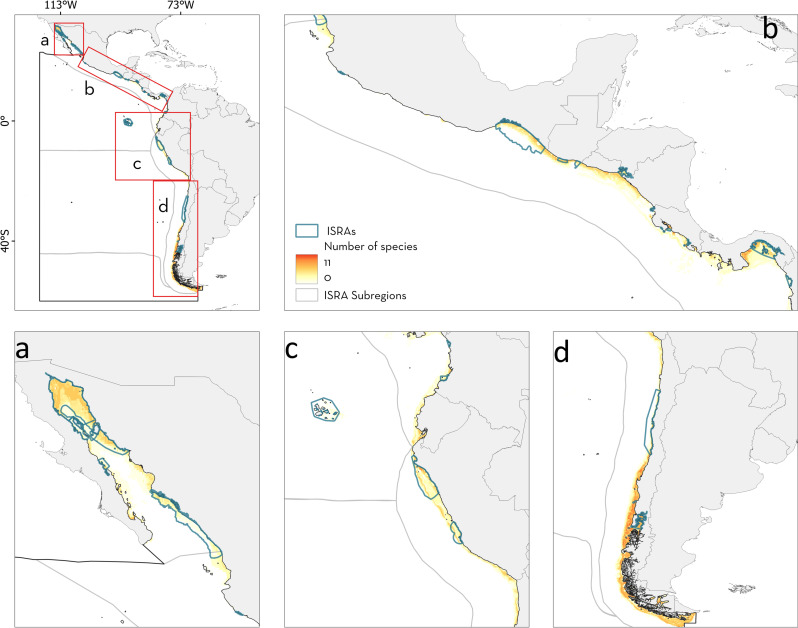
Spatial overlap between Important Shark and Ray Areas (ISRAs) identified under Criterion B - Range Restricted with areas of high richness for range-restricted species in the Central and South American Pacific region. (a) Gulf of California, (b) Pacific Central American Coastal, (c) southern part of Pacific Central American Coastal and northern part of Humboldt Current, and (d) Humboldt Current.

Identified ISRAs covered 34.40% of the total surface area where at least one range-restricted species was distributed in the region. In addition, ISRAs identified only for Criterion B - Range Restricted covered 26.85% of the total area where range-restricted species occur (Fig. 5). Areas with high species richness for range-restricted species (where >5 species occur) were delineated as ISRAs under Criterion B - Range Restricted (e.g., in the Gulf of California, southeast Mexico, Panama, and northern Peru). However, there was not enough information available to apply Criterion B - Range Restricted to several range-restricted species richness areas in Central America, southern Peru, and southern Chile (Fig. 5).

**Fig 6:**
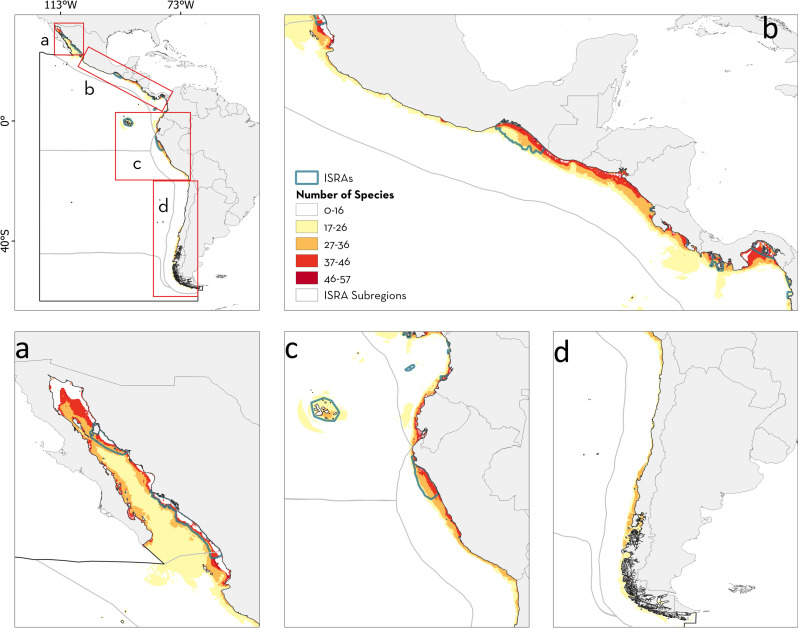
Spatial overlap between Important Shark and Ray Areas (ISRAs) identified under Sub-criterion D2 - Diversity with diversity hotspots in the Central and South American Pacific region. (a) Gulf of California, (b) Pacific Central American Coastal, (c) southern part of Pacific Central American Coastal and northern part of Humboldt Current, and (d) Humboldt Current.

For diversity hotspots, ISRAs covered 38.17% of the total area where more than 17 species are found. In addition, 17.20% of areas with >17 species present were delineated under Sub-criterion D2 - Diversity (Fig. 6). Delineated areas covered major richness areas in Colombia, Panama, northern Peru, and southeast Mexico. However, areas with the highest richness reported for the region (>45 species) in coastal Ecuador, northern Central America, and the northern Gulf of California were not identified as ISRAs this sub-criterion (Fig. 6). The northern Gulf of California was fully included in an ISRA supported by other criteria while the diversity hotspot in northern Central America was partially covered by small ISRAs.

### Research trends and gaps analysis

**Fig 7:**
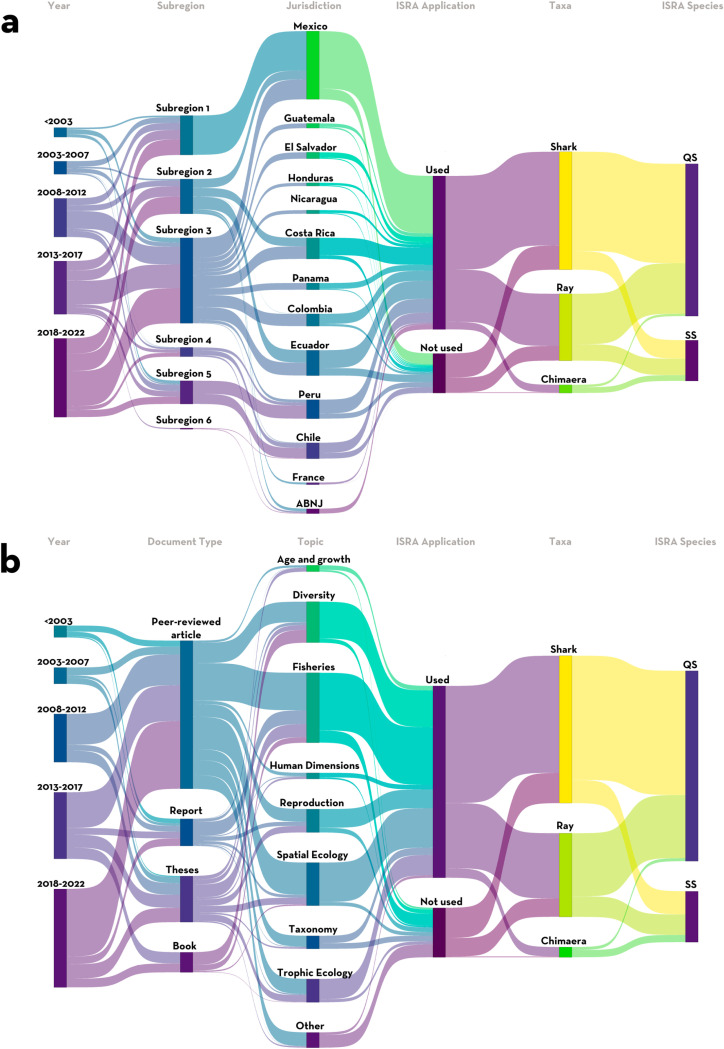
Reference analysis for contemporary (<20 years old) shark, ray, and chimaera research in the Central and South American Pacific region. (a) Spatial trends (b) Topic research trends. QS: Qualifying Species, SS: Supporting Species, ABNJ: Areas Beyond National Jurisdiction.

Based on the analysis of research outputs, 821 studies were found for chondrichthyans in the Central and South American Pacific region. Due to the multi-focus research topics of many studies, 2,106 entries were included in the analysis. An increasing trend in studies focusing on chondrichthyan research was noted in the last 20 years (i.e., since 2002), with 40.83% of references generated in the past five years (since 2017) and 68.38% generated in the past ten years (since 2012) Fig. 7). Research was primarily undertaken on sharks (n = 1,286 entries, 61.06%), followed by rays (n = 730, 34.66%), and chimaeras (n = 89, 4.23%; Fig. 7). Almost 80% of the entries (n = 1,675) were used as part of the ISRA process, either to support the inclusion of Qualifying Species (n = 1,348, 80.48%) or Supporting Species (n = 444, 19.52%; Fig. 7).

The Pacific Central American Coastal subregion (n = 931; 44.21%) had the most reference entries associated with it, followed by the Gulf of California (n = 430; 20.42%), Offshore Eastern Pacific (n = 379, 18%; Fig. 7a), and Humboldt Current (n = 253, 12.01%). Together, the Southeast Pacific subregion (n = 101) and the South American Southern Ocean (n = 12) had 5.37% of entries.

Mexico (n = 744; 35.33%) and Ecuador (n = 276; 13.11%) were the jurisdictions with the largest number of reference entries, followed by Costa Rica (n = 230; 10.92%), Peru (n = 207; 9.83%), Chile (n = 173, 8.21%), and Colombia (n = 134, 6.36%; Fig. 7a). Guatemala, Nicaragua, Honduras, El Salvador, and France contributed an aggregate of 10.26% (n = 216) of all the entries analyzed. Only 2.52% of research entries were from studies in areas beyond national jurisdiction.

Peer-reviewed articles were the most common type of reference (n = 510, 61.40%), followed by theses (n = 158, 19.09%). Peer-reviewed articles followed a similar temporal trend, while other references were equally produced in the past two decades (Fig. 7b).

The most common research topic was related to fisheries (n = 610, 28.96% of the entries), primarily focused on the description of catches and landings. Spatial ecology (n = 376, 17.85%) and diversity (n = 357, 16.95%) were dominant research topics, followed by reproductive biology (n = 204, 9.69%) and trophic ecology (n = 204, 9.69%). Age and growth (n = 56, 2.66%) and human dimensions (n = 53, 2.52%) studies were the least common topics (Fig. 7b).

## Discussion

Our analysis highlights how the wide variety of research outputs produced in the Central and South American Pacific region allowed the delineation of important habitats for chondrichthyans. This provides an opportunity to advance the conservation of these species by informing and guiding policy related to spatial planning and fisheries management. However, despite an increase in relevant scientific outputs since 2017, research has not equally focused on all species and topics, and habitats have not been equally studied. Here, we discuss the habitat and taxonomic representation in identified ISRAs, regional chondrichthyan research trends, and key knowledge gaps in relation to informing area-based management.

### Habitat and taxonomic representation

The 65 ISRAs identified within the Central and South American Pacific region expose spatial gaps and biases in knowledge. The majority of ISRAs (87.69%) were delineated in the northern part of the region, mostly in Mexico, Ecuador, and Costa Rica. Limited spatial and ecological information prevented the delineation of a larger number of ISRAs in countries such as Guatemala, El Salvador, Honduras, Nicaragua, and Panama. Coastal areas along with oceanic islands in the tropics (e.g., Galápagos Platform ISRA [21]) also had more data available. In fact, offshore areas in all jurisdictions were poorly represented and major spatial gaps were found in southern Chile, the oceanic islands in subtropical and temperate areas (e.g., Desventuradas Islands and Juan Fernández Archipelago), and in ABNJ. This appears to be directly linked to the production of scientific outputs as more references from northern subregions (78%) were available, and used within the ISRA process, compared to southern subregions. The limited knowledge and scientific output from these areas may be due to several reasons. Firstly, many of the large marine megafauna or ‘charismatic species’ that often receive a disproportionate amount of funding and research (e.g., Whale Shark, hammerhead sharks [Sphyrnidae], White Shark *Carcharodon carcharias*, or devil rays and mantas [Mobulidae]) have not been reported from southern subregions [31]. This has led to research effort on chondrichthyans being limited in southern subregions, likely due to resource allocation and funding that has been prioritized for large megafauna rather than poorly known chondrichthyan species. This is also often exacerbated by access to funding that is sometimes limited for low-income countries and non-English speakers [15,33,34]. Finally, in areas where large chondrichthyans fisheries exist and where data could be accessible (e.g., for Yellownose Skate *Dipturus chilensis* or Roughskin Skate *D. trachiderma* in Chile), market requirements can prevent biological sampling (N. Morales pers. obs. 2024). This has led to situations where ISRAs could not be identified for some regions. For example, southern Chile is characterized by high endemism and a high diversity of range-restricted species [23] but data were not available. Prioritizing research in such locations is important to ensure that key habitats are understood and can be considered in conservation planning.

Global patterns of knowledge highlight that coastal areas from tropical and subtropical regions are more commonly studied than oceanic regions and temperate and subpolar regions [15,35]. Similarly, although identified ISRAs cover a range of habitats, most were found on the continental shelf and in coral reefs (e.g., Cabo Pulmo ISRA), mangrove forests (e.g., Gulf of San Miguel and Tuira River ISRA), and seagrass beds (e.g., northern Gulf of California ISRA [21]). These habitats are globally recognized for their critical role in the life-history of many shark and ray species [36–39]. Coastal habitats are also those that have already been most impacted by anthropogenic activities (e.g., habitat degradation, pollution [36]). These need to be prioritized for conservation planning to conserve coastal species that are often those most threatened globally [40].

Poorly studied but important habitats such as oceanic and deep waters [41–43] were less represented in the ISRA process. Similarly, even though most globally distributed families (77%) were included in an ISRA, taxonomic gaps were evident for families with species associated with deepwater habitats (e.g., Arhynchobatidae, Centrophoridae, Dalatiidae). The remoteness of many of the habitats they occupy (e.g., Easter Island) make them logistically and economically challenging to monitor. This hinders long-term-studies to understand how chondrichthyans regularly and predictably use them (as required to apply the ISRA Criteria [2]). For example, while seamounts have been reported as important habitats for pelagic species, including sharks [44–46], few seamounts (n = 7) were proposed as ISRAs in the region (e.g., Cocos Island and Seamounts ISRA, Paramount Seamount ISRA [21]). In these cases, the standardized monitoring provided sufficient ecological data to successfully delineate critical habitats [47]. Further, even though ISRAs were identified in deepwater areas (>200 m; e.g., Northern Galápagos Hydrothermal Vents ISRA, Deep Benthic Midriff Islands ISRA [21]), many habitats where deepwater species commonly occur and that are well-distributed within the region, such as trenches, knolls, troughs and ridges (e.g., the Salas y Gómez and Nazca ridges [48]) were poorly represented due to lack of data.

Along the same lines, it is well established that research on chondrichthyans has commonly been dominated by a focus on sharks [9,13,49,50], specifically species in the order Carcharhiniformes. Although sharks and rays were equally included as Qualifying Species, chimaeras were underrepresented. Overall, these species have received less research focus than sharks and rays due to challenges in their identification, their distribution in deep waters, and their low economic value [9]. There were few contemporary observations for most deepwater species. This is likely because they have received limited attention and are not commonly caught or reported by fisheries [51]. However, research and monitoring of deepwater habitats and the species living in them is warranted [51]. Fishing fleets are expanding their operations to deeper waters and catching a larger number of species [52,53]. As a result of these expanding fisheries targeting chondrichthyans for their oil and meat, drastic population declines have been reported globally (e.g., gulper sharks [Centrophoridae] [51]). Furthermore, with the growing interest in deep-sea mining worldwide, there are concerns that the extraction of minerals will result in significant loss of biodiversity, as well as major impacts on deepwater ecosystems [54]. As new technologies (e.g., remotely operated underwater vehicles) become more common to explore deepwater areas, the availability of information needed to identify these important habitats [55–57] is likely to increase.

### ISRA criteria applied

Most threatened species found in the region (59 of 75 species) were included as Qualifying Species in at least one ISRA. This is a reflection of the elevated extinction risk of chondrichthyans globally [6]. However, an ISRA cannot be delineated solely based on the presence of threatened species; an additional criterion linked to the species’ specific use of that habitat must be applied [2]. ISRAs delineated for threatened species (46%) were characterized by key habitats for reproductive or feeding purposes. Information available to identify reproductive areas for sharks and rays has benefited from the recognition of nursery areas as critical habitats through the definition and application of criteria [58–60]. This led to ongoing research efforts across the region to identify such areas which in turn increased the information available to apply the ISRA Criteria. Such information was frequently supplemented with fisheries studies (e.g., [61,62]). For example, body size estimates (i.e., total length for sharks or disc width for rays) derived from fisheries catch and landings data were critical to confirm the regular presence of early-life stages and/or the presence of pregnant females in several areas (e.g., Costa Chica of Oaxaca ISRA, Santa Elena Gulf ISRA [21]). While such life-history processes can be some of the more easily identified aspects in terrestrial fauna (e.g., [63]), gathering robust evidence to delineate boundaries can be challenging in aquatic ecosystems. Therefore, the use of complementary approaches (fishery-dependent and independent) to collect data is encouraged. This can also lead to the delineation of smaller areas that are truly required for species’ recovery [63]. Continuous data collection using these methods is necessary to ensure monitoring of these sites. Finally, researchers should be encouraged to gather spatially explicit information on fishing locations that can help further refine boundaries of important areas (e.g., [64]). It is acknowledged that spatial information is not always possible to gather as fishers can be reluctant to share it [65] and monitoring is mostly conducted at landing sites. In such cases, incorporating local ecological knowledge and human dimensions (e.g., involvement of fishing communities in data collection) can support in the delineation of important habitats (e.g., Humboldt Current and Transition Zone ISRA [66]).

Movement Areas were mostly delineated in subregions 2 and 3 (e.g., Eastern Tropical Pacific Marine Corridor ISRA; Jabado et al. 2023) and were correlated with the increase in the use of acoustic and satellite tags [67,68]. The establishment of large tracking consortiums exploring the movement of animals and connectivity between oceanic islands and coastal areas, mainly in Mexico, Costa Rica, Colombia, and Ecuador has played a key role in data availability [67,69,70]. This was reflected in the number of entries for movement studies (‘Spatial Ecology’; n=376) which revealed the presence of important migratory corridors for sharks and rays [70,71]. Movement areas often cover large surface expanses due to the scale of seasonal migrations that some species undertake (e.g., Whale Shark [72]) and are key to highlight ecological connectivity. Such areas were not identified in the southern part of the region, due to a lack of information rather than habitat use. Tracking data for chondrichthyans is limited overall [73], but with the increase in such studies, it is likely that additional Movement Areas will be delineated in future. The effective management and conservation of large migratory corridors can be challenging, and the enforcement of legislation at such levels can be particularly difficult for smaller nations, which often have a disproportionately large ocean territory relative to their landmass [74]. Although ISRAs for important Movement Areas in coastal habitats (Costa Rica - Cabo Blanco ISRA [65.6 km^2^], Coyote-Bongo ISRA [95.42 km^2]^ [21]) may be easier to consider for conservation planning, the creation and expansion of large MPAs based on the movement of sharks and rays in the Eastern Tropical Pacific has been possible (Revillagigedo, Cocos, Malpelo, and the Galápagos [67,71]). Some of these have been very successful, although challenges in enforcement have been highlighted [75,76]. Ensuring the effectiveness of these MPAs will require collaboration at the regional scale. On the other hand, the uptake of smaller ISRAs into area-based management is likely to provide more benefits to some species as smaller protected areas can conserve a large portion of a species’ range compared to highly migratory species [77]. Overall, ISRAs delineated as important movement areas can be used in both coastal and pelagic waters to inform spatial-based management across the region.

### Chondrichthyan research trends and opportunities to fill existing knowledge gaps

Globally, the volume of peer-reviewed chondrichthyan research is growing exponentially, with the number of scientific papers published doubling each decade [6,49]. The same pattern was observed in this region with the doubling of research outputs in the last five years. Although the majority of research outputs analyzed were peer-reviewed articles, grey literature (theses and technical reports), and local ecological knowledge were important sources of evidence to support the identification of ISRAs. Collating and incorporating all data sources is critical to explore population trends [78] and identify critical habitats [66,79,80]. Research outputs most useful to support the identification of ISRAs were traditional research (e.g., age and growth) and studies incorporating more recent technologies (e.g., satellite and acoustic telemetry) provided strong evidence. However, many studies lacked key data due to the methodology limitations. For example, some photo-identification studies showed connectivity between areas, but information was not available on the specific movements of individuals [81]. Similarly, research design often omitted key information that could have been used to identify critical habitats. For instance, feeding studies often described specific prey and temporal patterns of feeding but did not relate it to environmental features that trigger this process at the site level [82]. We encourage future research to take into consideration the need to collect data to support the delineation of critical habitats and combine complementary methodologies (e.g., photo-identification with telemetry data).

Despite Mexico and Ecuador being the jurisdictions with the largest number of studies in the region, spatial research gaps were still identified. While monitoring in jurisdictions with low-research outputs should be prioritized, it could also be improved in high research effort locations (e.g., Gulf of California and the Galápagos Islands). Research at these sites has focused on a small number of the species occurring there likely due to funding constraints limiting the scope of research. Resources in these areas should be reallocated toward other species, particularly threatened and often overlooked ones. Large chondrichthyans with a wide distribution and occupying surface and coastal waters have attracted considerably more research than small species [13]. This holds true for the species included in a larger number of ISRAs (e.g., Scalloped Hammerhead, Oceanic Manta Ray). These species utilize coastal areas during part of their life cycle, are considered ‘charismatic species’ [13], contribute to ecotourism activities [83,84], and/or are commonly captured in fisheries [85]. This increases public interest in them and attracts larger research budgets [50,86,87]. Consequently, most of the important habitats for their life-history processes have been well studied and identified. For some species (e.g., Whale Shark, Oceanic Manta Ray), the relatively high-level of knowledge along with their high extinction risk have led to national protection in most of the region (e.g., Mexico [88]). These species could withstand a decrease in research effort without diminishing their conservation benefits. This would allow to balance the research focus on poorly studied, under-represented, and threatened species [51,89] for which important habitats have yet to be identified.

The most threatened families globally (pelagic eagle rays [Aetobatidae], devil rays [Mobulidae], sawfishes [Pristidae], hammerheads [Sphyrnidae] and angel sharks [Squatinidae] [6]) were included in ISRAs. Their inclusion as Qualifying Species was due to an increase in research effort in the last ten years [82,90,91] likely a reflection of their elevated extinction risk and the need for data to improve their management and conservation. As important habitats have been identified for these species, mechanisms should be put in place to properly manage and protect them from existing threats and support population recovery. Particular focus could be given to critical habitats identified for species extirpated across most of historical distributional range in the region such as sawfishes [92] in the Gulf of Montijo ISRA in Panama [21].

Due to the scarcity of biological and ecological data, important habitats could be identified for a small proportion of species assessed as Least Concern (20%) and Data Deficient (7%). While for Least Concern species current threat levels do not pose a major risk, Data Deficient species need to be prioritized. A large proportion of these species are likely to be threatened since they often share life-history traits with other threatened species and are likely exposed to similar fishing pressure [6]. Research should focus on all Data Deficient species but prioritize those that are endemic to this region such as the Roughskin Eagle Ray *Aetomylaeus asperrimu*s, Eastern Pacific Black Chimaera *Chimaera orientalis*, Ecuador Skate *Dipturus ecuadoriensis*, and Spotted Round Ray *Urobatis marmoratus*. Resource allocation will continue to be a challenge in this region, but strategic and cost-effective monitoring of certain species could serve a proxy for a suite of other species and therefore inform their management and conservation.

### Relevance of ISRAs for area-based management

As 2030 approaches, Parties will need to deliver on their commitment under Target 3 of the Global Biodiversity Framework to protect 30% of inland and marine waters [19]. Across the Central and South American Pacific region, only Panama and Chile have reached these targets [19]. Further, while MPA designation in the region quickly increased after 2010, most protected areas do not overlap with critical habitats for chondrichthyans, limiting the conservation benefit for these species [93]. ISRAs provide an opportunity for governments to advance conservation for these species while contributing to meet global targets. If these ISRAs are effectively managed, almost 100 species will benefit and vital life-history functions for threatened and endemic species along with diversity hotspots can be protected in national waters and in ABNJ. ISRAs also present an opportunity to not only design and delineate new protected areas but to improve existing MPAs where an overlap occurs [93]. Many of the existing protected areas do not have a management plan or do not recognize chondrichthyans in them [93]. In such cases, ISRAs can be used to inform zonation in existing MPAs to secure the conservation of these species within their boundaries. Finally, fishing mortality is the main threat to chondrichthyans [6]. Effective enforcement of fishing regulations may be challenging, especially for artisanal and multi-species coastal fisheries which are widespread in the region [94]. ISRAs provide an opportunity to improve fisheries management actions that can reduce mortality and can be used to prioritize areas to focus enforcement and monitoring too [2,93]. This can lead to improved compliance of existing regulations in critical habitats for these species, including the retention ban for threatened species in fisheries regulated by regional fisheries management organizations (e.g., Whale Shark and devil rays in the Inter American Tropical Tuna Commission [IATTC] [95,96]), retention limits (e.g., Silky Shark in IATTC convention area [97,98]), or seasonal bans (e.g., Mexican Pacific [99]).

### Final considerations

Despite existing research gaps, the current state of knowledge of sharks, rays, and chimaeras in the Central and South American Pacific region has allowed the delineation of ISRAs covering the majority of species, including the most imperiled ones (e.g., sawfishes, devil rays, hammerheads). This highlights that there are sufficient data available to inform area-based management including the design of MPAs or fisheries spatial measures (e.g., fishing closures, gear restrictions) that can help reduce existing threats and advance the conservation of these species. An increase in funding and ecological research with spatial components focused on poorly studied habitats and species is needed to successfully map critical habitats that could be protected to secure the conservation of all chondrichthyan species in this region. Overall, while there remain challenges in identifying ISRAs for some species, especially in locations where gaps in biological and ecological knowledge exist, actions taken within ISRAs are encouraged and are likely to also benefit the less studied species that occupy these habitats. This will be key to ensure the long-term survival of these species.

## Supporting information

S1 Fig Spatial distribution of Important Shark and Ray Areas (ISRAs) identified for Criterion C - Life-history in the Central and South American Pacific. Sub-criterion C1 - Reproduction, Sub-criterion C2 - Feeding, Sub-criterion C3 - Resting, Sub-criterion C4 - Movement, Sub-criterion C5 - Undefined Aggregations.
(PNG)

S1 Table Number of ISRAs per Criteria met by each shark, ray and chimaera species occurring in the Central and South American Pacific region. (CSV)

S2 Table List of research outputs in the Central and South American Pacific region. (XLSX)
