# Supplementary figures and images for: Mapping Important Shark and Ray Areas (ISRAs) in the Central and South American Pacific: Existing knowledge and data needs
Source: PLoS One. 2025 May 7;20(5):e0322445. doi: 10.1371/journal.pone.0322445 (PMC12058020; doi:10.1371/journal.pone.0322445)

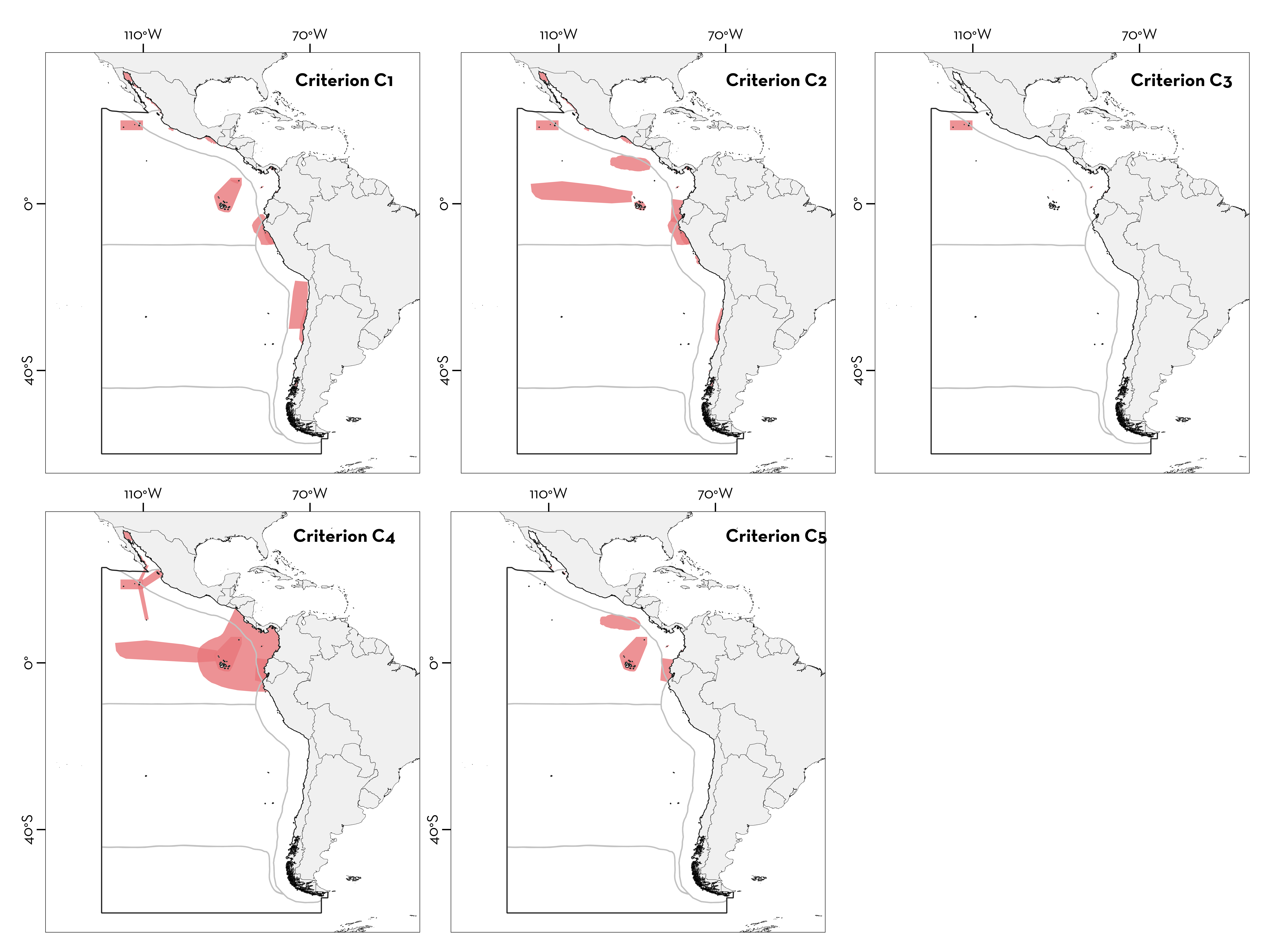

Supplement: S1 Fig — Sub-criterion C1 - Reproduction, Sub-criterion C2 - Feeding, Sub-criterion C3 - Resting, Sub-criterion C4 - Movement, Sub-criterion C5 - Undefined Aggregations. (PNG) [file pone.0322445.s001.png]
